# Mid-Term Clinical, Radiographic, and Safety Outcomes After Hemiarthroplasty with the CL TRAUMA Cemented Femoral Stem: A Post-Market Clinical Investigation

**DOI:** 10.3390/jcm15218640

**Published:** 2026-11-06

**Authors:** Carlo Biz, Assunta Pozzuoli, Giacomo Maso, Elisa Belluzzi, Pietro Ruggieri

**Affiliations:** 1 Orthopedics and Orthopedic Oncology, Department of Surgery, Oncology and Gastroenterology, University-Hospital of Padova, 35128 Padova, Italy; carlo.biz@unipd.it (C.B.); assunta.pozzuoli@unipd.it (A.P.); giacomo.maso@aopd.veneto.it (G.M.); pietro.ruggieri@unipd.it (P.R.); 2 Centre for Mechanics of Biological Materials, University of Padova, 35131 Padova, Italy; 3 Musculoskeletal Pathology and Oncology Laboratory, Department of Surgery, Oncology and Gastroenterology, University of Padova, 35128 Padova, Italy

**Keywords:** femoral neck fracture, hemiarthroplasty, cemented femoral stem, hip arthroplasty, implant safety, level of evidence

## Abstract

**Background/Objectives**: Cemented femoral stems are a well-established treatment option for femoral neck fractures (FNFs) in elderly patients. This study evaluated mid-term clinical and radiographic outcomes and safety profile in patients undergoing hemiarthroplasty with the CL TRAUMA cemented femoral stem (LimaCorporate) for FNF. **Methods**: The single-center observational Post-Market Clinical Investigation identified 474 patients who underwent hip arthroplasty between 2017 and 2023. Of these, 54 (11.4%) who were alive, contactable, willing, and physically able to attend were included in the mid-term follow-up (FU) assessment. Perioperative, discharge, and 1-month FU data were collected retrospectively, while mid-term clinical and radiographic FU was performed prospectively after a minimum of 2 years. The primary endpoint was satisfactory status, defined as a Harris Hip Score (HHS) ≥ 80. Secondary endpoints included patient satisfaction assessed using Visual Analogue Scale (VAS), radiographic outcomes, and documented serious adverse events (SAEs). **Results**: The 54 evaluated patients (54/474; 11.4% of the implanted population) included 49 females and 5 males, with a mean age at surgery of 77.1 ± 9.1 years and a mean FU of 4.4 ± 0.8 years. All patients underwent hemiarthroplasty for FNF. Mean HHS was 80.6 ± 16.9, with 33 patients (61.1%) achieving the primary endpoint. Mean VAS satisfaction was 76.4 ± 29.3. At mid-term FU, RLLs were observed in 38.9% of patients, predominantly of limited thickness. Distal stem migration was observed in one case (1.9%), and bone remodeling in three patients (5.6%). Eleven SAEs occurred in 10 patients (18.5%); three events were related and two possibly related to the surgical procedure, whereas none was considered related to the device. No femoral stem revisions were documented among the 54 evaluated patients during the available FU. **Conclusions**: Among the selected patients who survived and completed mid-term follow-up after bipolar hemiarthroplasty with the CL TRAUMA cemented femoral stem, satisfactory functional status and patient satisfaction were observed, together with generally stable stem-specific radiographic findings. No SAEs were considered related to the femoral stem, and no femoral stem revisions were documented. These findings should be interpreted cautiously and cannot be extrapolated to the entire implanted population.

## 1. Introduction

Hip fractures represent one of the most common and challenging injuries in the elderly population and are associated with substantial morbidity, mortality, loss of independence, and deterioration in quality of life [1,2,3]. Their incidence continues to rise worldwide as a consequence of population aging and increased life expectancy, with projections estimating a marked increase in the number of cases over the coming decades [4,5,6]. Given the importance of preserving mobility and functional independence in older adults, as well as the severe consequences of prolonged immobilization, restoration of mobility remains a primary goal of treatment following hip fracture [2].

Among hip fractures, femoral neck fractures (FNFs) account for approximately half of all cases and represent a major indication for arthroplasty in elderly patients [7]. The choice of treatment depends on several factors, including fracture displacement, patient age, functional status, and comorbidities [8]. The Garden classification remains clinically relevant in elderly patients with FNFs, as non-displaced Garden I–II fractures are commonly considered for internal fixation and fracture morphology influences the risk of treatment failure [9]. For displaced FNFs (Garden III and IV), the two main surgical treatment options are hemiarthroplasty (HA) and total hip arthroplasty (THA) [2]. In elderly patients, HA is widely adopted because it provides reliable pain relief, facilitates early mobilization, and promotes recovery of hip function and independence, while reducing complications associated with prolonged immobilization [4,6,10]. Compared with THA, HA is associated with a less invasive procedure, shorter operative time, reduced blood loss, and lower costs, making it an attractive option for elderly and frail patients [6]. However, the management of these patients remains challenging, as they frequently present with osteoporosis, multiple comorbidities, and an elevated perioperative risk. Despite advances in perioperative care, mortality following hip fracture remains high, with one-year mortality rates reported to exceed 25% in elderly patients, highlighting the importance of optimizing surgical strategies and implant selection to improve outcomes [1].

Among the factors influencing outcomes after HA, the method of femoral stem fixation remains a topic of ongoing debate. Cemented femoral stems are commonly used in elderly patients because they provide immediate implant stability and reliable fixation, particularly in osteoporotic bone, and have been associated with lower rates of postoperative thigh pain and periprosthetic fractures compared with uncemented implants [6]. Nevertheless, concerns have been raised regarding bone cement implantation syndrome (BCIS), cardiopulmonary complications, and the technical challenges associated with revision surgery [11]. Advances in implant design, biomaterials, and cementing techniques, including improved femoral canal preparation and modern polymethylmethacrylate (PMMA)-based cements, have contributed to enhancing implant fixation and reducing complication rates [5,6].

In accordance with the European regulatory framework for medical devices (2017/745), post-market clinical investigation is intended to continuously update the clinical evaluation of a device by collecting and assessing clinical data after market introduction. For implantable devices used in hip arthroplasty, such post-market evidence is particularly relevant to confirm safety, clinical performance, radiographic stability, and safety in routine practice. Therefore, post-market evaluation of cemented femoral stems is essential to evaluate their safety, radiographic and clinical performance, particularly in elderly patients with FNFs. The CL TRAUMA femoral stem is a monolithic cemented stem intended for HA or THA, including the treatment of femoral head and neck fractures. Therefore, the aim of this study was to evaluate the mid-term clinical and radiographic outcomes of the CL TRAUMA cemented femoral stem in a cohort of patients undergoing HA for FNFs. Secondary objectives included assessment of implant safety and the incidence and characterization of adverse events (AEs).

## 2. Materials and Methods

### 2.1. Study Design

This was a single-center, observational post-market clinical investigation (PMCI) of the CL TRAUMA femoral stem, a CE-marked device used in accordance with its intended use. Intraoperative, discharge, and early postoperative follow-up (FU) data were collected retrospectively, whereas mid-term FU was performed prospectively through clinical and radiographic assessment.

The study was approved by the Institutional Ethics Committee (Approval Code: 5421/AO/22) and conducted in accordance with the ethical standards of both the Declaration of Helsinki (2024 revision) and the Good Clinical Practice guidelines. It was registered on ClinicalTrials.gov (registration number: NCT05606120, first posted 4 November 2022), after the beginning of the historical implantation period in 2017 but before prospective patient enrollment, which began on 14 March 2023.

The approved study protocol included retrospective screening of all patients who had received the CL TRAUMA femoral stem, followed by telephone contact with potentially eligible patients to invite them to participate in the mid-term FU assessment. As part of this screening process, vital status and, where applicable, dates of death were ascertained for the original implanted population (*n* = 474). These data, together with the dates of the index surgical procedures, were used to estimate overall patient survival using the Kaplan–Meier method.

Detailed clinical, radiographic, and safety outcomes were analyzed only for the patients formally enrolled in the study, all of whom provided written informed consent for participation and the processing of their personal data.

### 2.2. Patients

Consecutive patients who underwent hip arthroplasty with the CL TRAUMA femoral stem between January 2017 and January 2023 were retrospectively identified from the institutional database. Eligible patients were prospectively enrolled between March 2023 and January 2025 and invited to attend the mid-term FU visit.

Eligibility criteria were defined according to the approved indications for use of the CL TRAUMA femoral stem and therefore encompassed both HA and THA, as appropriate for the underlying clinical indication. Inclusion criteria were (i) age > 18 years; (ii) a clinical indication for hip arthroplasty with the CL TRAUMA femoral stem, including non-inflammatory degenerative joint disease, rheumatoid arthritis, or femoral head and neck fracture; (iii) written informed consent for study participation; and (iv) ability to comply with the study protocol and attend the mid-term FU assessment.

Exclusion criteria were (i) active or suspected local or systemic infection; (ii) acute or chronic osteomyelitis; (iii) confirmed neuromuscular disorders compromising hip joint function; (iv) vascular or neurological diseases affecting the involved limb; (v) poor bone stock or extensive previous revision surgery compromising implant stability; (vi) metabolic disorders potentially impairing implant fixation and stability; (vii) concomitant conditions or dependencies potentially affecting prosthesis performance; (viii) known hypersensitivity or allergy to implant materials; (ix) participation in other surgical intervention or pain-management studies; and (x) pregnancy, breastfeeding, or planned pregnancy.

### 2.3. Implant Device

**Figure 1:**
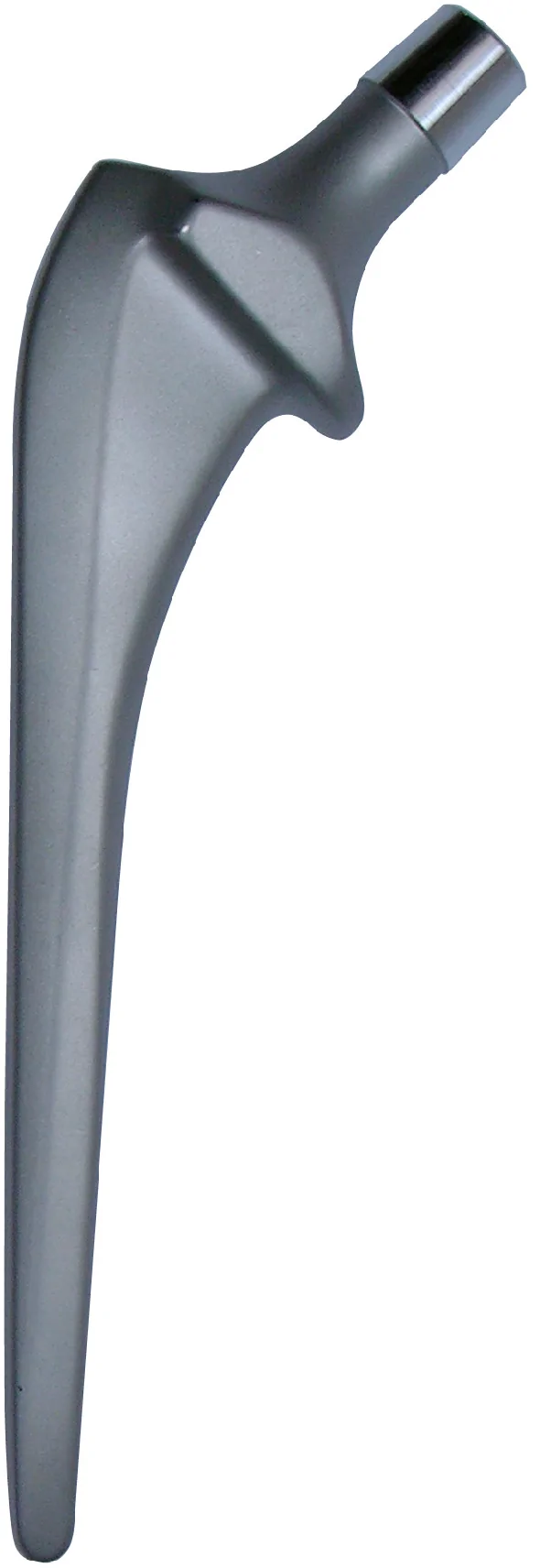
CL TRAUMA femoral stem. Courtesy of LimaCorporate S.p.A.

The CL TRAUMA femoral stem (LimaCorporate S.p.A., Villanova di San Daniele del Friuli, Udine, Italy) is a long-term implantable Class III medical device according to Directive 93/42/EEC and European Directive 2005/50 CE (Figure 1). It is a monolithic cemented femoral component designed to optimize femoral canal filling and cement distribution, thereby enhancing implant stability, even in compromised bone conditions. The stem features a straight design with a large collar, a 135° CCD angle, and is manufactured from AISI 316L stainless steel. The 12/14 Morse taper is compatible with femoral and bipolar heads ranging from 22 to 36 mm, allowing its use in both HA and THA procedures according to the device indications for use.

### 2.4. Surgical Technique

Surgical procedures were performed within 48 h from admission by three experienced orthopedic surgeons using the surgical approach deemed most appropriate based on patient characteristics, fracture pattern, and surgeon judgement. The anterolateral (Watson–Jones), direct lateral (Bauer), and posterolateral (Moore) approaches were used.

Following surgical exposure, capsulotomy and, when appropriate, partial or complete capsulectomy were performed. Femoral neck osteotomy was followed by removal of the femoral head, which was measured to select the appropriate prosthetic head size. The femoral canal was subsequently prepared using dedicated instruments, including progressively sized broaches, according to the manufacturer’s recommendations. Femoral bone stock quality was qualitatively assessed intraoperatively by the operating surgeon and classified as normal or porotic based on the perceived mechanical resistance of the bone during femoral preparation. Following canal preparation, femoral canal brushing and lavage were performed to remove residual bone debris and marrow contents before cementation.

Trial components were used, as appropriate, to determine the optimal implant size and assess leg length, femoral offset, joint stability, and soft-tissue tension before definitive implantation. Bone cement was prepared according to a standardized protocol using a vacuum-mixing system. Non-antibiotic-loaded Simplex P Bone Cement (Stryker; reference number 6191-0-001; medium-to-low viscosity) was used in all procedures. A distal intramedullary cement restrictor was positioned before cementation of the femoral canal and insertion of the definitive CL TRAUMA femoral stem. After implantation, the definitive femoral head was inserted and the joint was reduced. Intraoperative assessment included evaluation of implant stability, range of motion (ROM), leg length, and soft-tissue tension through passive mobilization and stability testing. Final intraoperative fluoroscopic control was performed in all procedures.

Following confirmation of implant stability, meticulous hemostasis and irrigation were performed. Where applicable, the joint capsule and detached musculotendinous structures were repaired. Suction drains were placed according to surgeon preference, followed by layered wound closure.

Postoperatively, patients followed a standardized rehabilitation protocol. A physiotherapy assessment was performed on the first postoperative day to guide early mobilization and define an individualized rehabilitation program. Patients were encouraged to stand and mobilize as soon as clinically feasible, followed by progressive muscle strengthening and gait training according to their clinical condition, functional recovery, and tolerance to rehabilitation.

### 2.5. Clinical Assessment

Demographic, surgical, and postoperative data were collected retrospectively from medical records. Additional preoperative and perioperative variables, including ASA physical status, time from hospital admission to surgery, length of hospital stay, perioperative blood transfusions, and discharge destination, were retrospectively extracted from the available medical records. History of osteoporosis was derived from available medical records and patient-reported medical history.

Study visits and corresponding time windows were predefined. Intraoperative, discharge, and 1-month FU data were retrieved retrospectively. The intraoperative assessment occurred on the day of implant surgery, while the discharge visit followed standard clinical practice at our institution. The 1-month FU visit was scheduled at 1 month (±30 days) postoperatively.

Eligible patients identified through database screening were subsequently contacted and invited to participate in a mid-term FU visit. This visit was conducted prospectively to assess implant safety and mid-term clinical and radiographic outcomes, as well as general health status, medical history, overall well-being, and satisfaction with the prosthesis.

Clinical evaluation at the mid-term FU visit included the Harris Hip Score (HHS) and a Satisfaction Visual Analogue Scale (VAS). The HHS comprises four domains: pain (44 points), function (47 points), ROM (5 points), and absence of deformity (4 points). Results are classified as “excellent” (90–100), “good” (80–89), “fair” (70–79), “poor” (60–69), and “failure” (<60) [12]. An HHS ≥ 80 is considered a satisfactory outcome. The HHS was administered at the mid-term FU visit by an orthopedic surgeon who was independent of the operating surgical team.

Ambulatory status was further characterized using the gait-related components of the HHS, including limp severity, walking support, and walking distance.

Patient satisfaction was assessed using a 0–100 Visual Analogue Scale (VAS), where 0 indicated “no satisfaction” and 100 indicated “extreme satisfaction” [13].

### 2.6. Radiographic Assessment

Standard anteroposterior (AP) pelvis and lateral (LAT) hip radiographs were performed preoperatively, at discharge, 1-month FU, and at the final mid-term FU.

Preoperative radiographs were retrospectively reviewed to classify FNFs according to the Garden classification [14]. Fractures were categorized as Garden I (incomplete and valgus-impacted), Garden II (complete and nondisplaced), Garden III (complete and partially displaced), or Garden IV (complete and fully displaced). Other preoperative characteristics, including hip alignment, osteophytes, cysts, and sclerosis, were assessed.

Postoperative radiographic assessment included femoral stem positioning, RLLs, distal femoral component migration/subsidence, and bone remodeling. RLLs were evaluated on AP and LAT radiographs according to the Gruen zones, with their location and thickness recorded [15]. Longitudinal changes in RLLs were assessed by comparing their presence, width, and extent across successive radiographic evaluations.

Initial cement mantle quality was assessed on postoperative discharge radiographs according to the Barrack classification [16]. Grade A indicated complete filling of the medullary canal without RLLs at the cement–bone interface; grade B, radiolucent lines involving <50% of the cement–bone interface; grade C, RLLs involving 50–99% of the interface and/or an incomplete cement mantle; and grade D, complete radiolucency at the cement–bone interface and/or absence of cement distal to the stem tip.

Acetabular erosion was assessed according to the Baker classification [17]: grade 0, no erosion; grade 1, narrowing of the articular cartilage without bone erosion; grade 2, acetabular bone erosion with early migration of the prosthetic head; and grade 3, protrusio acetabuli.

Femoral stem subsidence was assessed on serial AP radiographs by evaluating distal migration of the femoral component relative to fixed anatomical landmarks of the proximal femur, as described in the literature [18]. Radiographs obtained at discharge and subsequent FU visits were compared, and any detectable migration was recorded in mm. A threshold of >5 mm was used to define relevant femoral stem subsidence [19].

Radiographic assessments were independently performed by two orthopaedic surgeons uninvolved in the surgical procedures and blinded to clinical outcomes. Inter- and intra-observer reliability, assessed using Cohen’s kappa and intraclass correlation coefficients (ICCs), ranged from 0.70 to 0.85, indicating moderate to good reproducibility.

### 2.7. Safety

Safety data were collected for the 54 patients enrolled in the post-market clinical investigation. Perioperative and early postoperative safety information was retrospectively retrieved from available medical records, whereas subsequent events were documented during the mid-term follow-up assessment. The observation period extended from the index surgical procedure to the final follow-up visit.

Safety monitoring included AEs, serious adverse events (SAEs), adverse device effects (ADEs), and device deficiencies (DDs), with particular attention to serious adverse device effects (SADEs) and unanticipated serious adverse device effects (USADEs). Although safety information was collected over the study period, only events meeting the criteria for SAEs were documented in the study dataset and included in the safety analysis. Documented SAEs were characterized according to their clinical description, timing, seriousness, severity, management, outcome, expectedness, and causal relationship with the investigational device and study procedure. The causal relationship of AEs to the investigational device and study procedure was assessed by the clinical investigators and documented in the study records. Femoral stem revision for any reason was predefined as the endpoint for implant survivorship. However, because systematic revision and mortality data were unavailable for the entire implanted population, a formal implant survivorship analysis was not performed.

### 2.8. Study Endpoints

The primary endpoint was the proportion of patients achieving an HHS ≥ 80 at mid-term FU. No formal performance success criterion, expressed as a predefined minimum proportion of patients achieving this threshold, was specified in the study protocol. Therefore, the primary endpoint was analyzed descriptively. Secondary endpoints included:

1. VAS satisfaction;

2. Radiographic assessment of implant stability, including RLLs, stem migration/subsidence, and bone remodeling;

3. Incidence and characterization of AEs, SAEs, SADEs, and ADEs.

### 2.9. Statistical Analysis

No formal sample size calculation was performed because this was a post-market observational study. All eligible patients who met the inclusion criteria and completed the mid-term FU assessment during the study period were included in the final analysis.

Descriptive variables and clinical outcomes were summarized according to data type. Continuous variables were reported using appropriate measures of central tendency and dispersion, whereas categorical variables were summarized as frequencies and percentages. The proportion of patients achieving the primary endpoint (HHS ≥ 80) was expressed as a percentage with its corresponding 95% confidence interval, calculated using the method by Agresti & Coull [20]. Two-sided 95% Student-t confidence intervals were calculated for the mean of continuous parameters. Two-sided 95%-Agresti & Coull confidence intervals were calculated for proportions of count parameters.

Overall patient survival was analyzed separately in the entire implanted cohort (*n* = 474) using the Kaplan–Meier method. The date of the index surgical procedure was considered the time origin, and death from any cause was defined as the event of interest. Patients confirmed to be alive at the study end date were administratively right-censored at that date. Survival probabilities at 30 days, 1 year, and 5 years were estimated with corresponding 95% confidence intervals. This analysis assessed patient survival and was distinct from femoral stem survivorship, which could not be reliably estimated for the overall implanted population. Kaplan–Meier survival analysis was performed using GraphPad Prism version 9.5.1 for Windows (GraphPad Software, LLC, San Diego, CA, USA).

An additional descriptive sensitivity analysis of operative time was performed by excluding two procedures involving additional surgical interventions to assess their influence on the overall operative-time distribution. Operative time was summarized using mean, standard deviation, median, interquartile range (IQR), and range.

Exploratory associations between HHS and age at surgery, BMI, and patient satisfaction assessed using the VAS were evaluated using Spearman’s rank correlation coefficient (ρ), with corresponding 95% confidence intervals. A total of three Spearman correlation tests were performed.

All statistical analysis was done with the free open-source software R, version 4.4.2 and using the packages “tidyverse”, “MASS”, and “psych” [21]. *p*-values less than or equal to 0.05 were considered statistically significant. No imputation for missing data was performed.

## 3. Results

### 3.1. Patients

**Figure 2:**
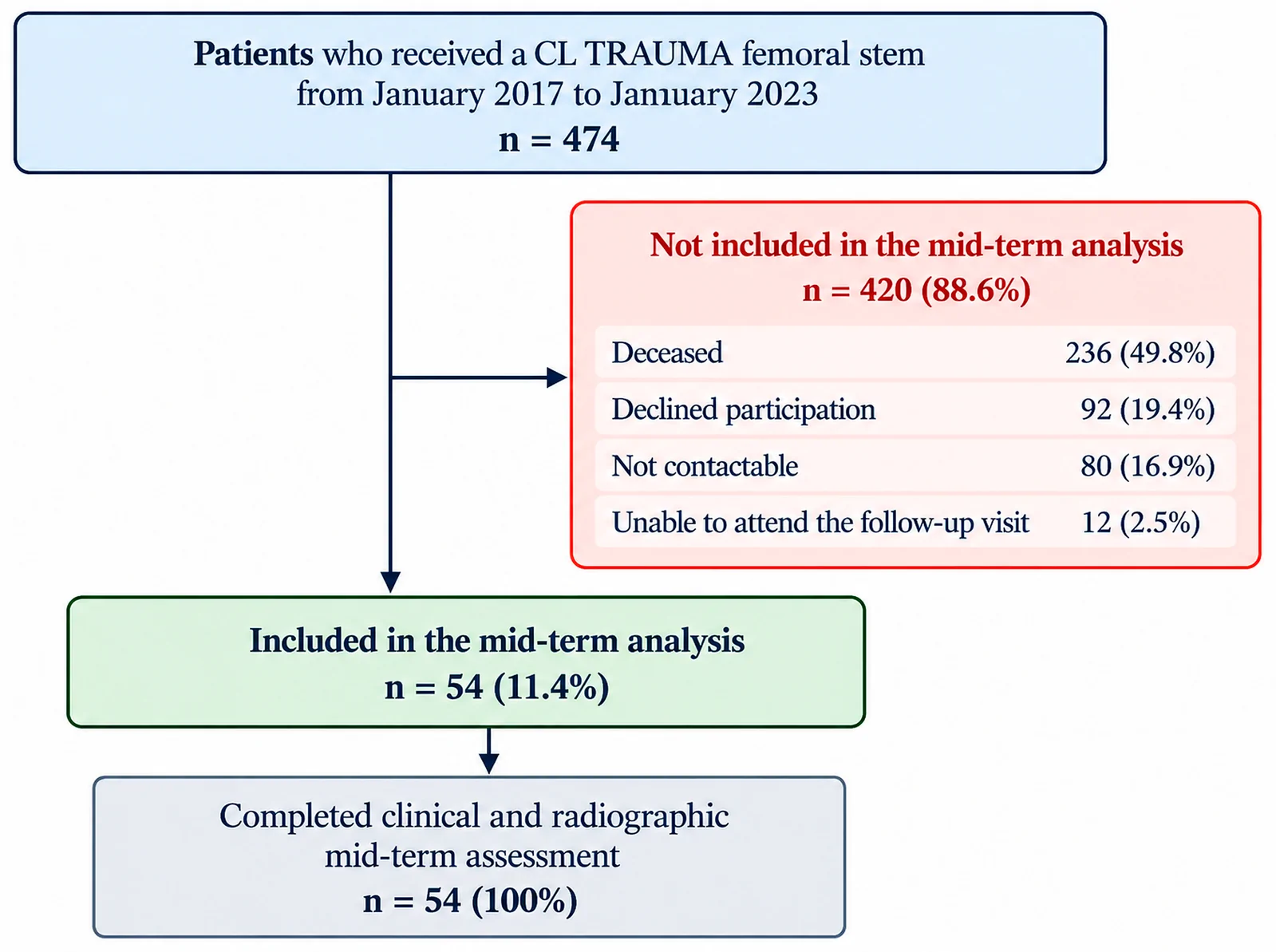
Flow diagram of patient screening and inclusion in the mid-term follow-up analysis.

Between January 2017 and January 2023, 474 patients underwent hip arthroplasty with the CL TRAUMA femoral stem and were screened for inclusion. Of these, 236 patients had died, 92 declined participation, 80 could not be contacted, and 12 were unable to attend the mid-term FU visit. Consequently, 54 of 474 implanted patients (11.4%) were available for evaluation and constituted the final study cohort (Figure 2).

The crude overall mortality was 49.8% (236/474 patients). In the overall implanted cohort (*n* = 474), Kaplan–Meier analysis showed an estimated 30-day mortality of 4.2% (95% CI: 2.7–6.5%). Estimated patient survival was 76.6% at 1 year (95% CI: 72.5–80.1%) and 53.2% at 5 years (95% CI: 48.4–57.8%) (Supplementary Figure S1).

All enrolled patients attended the mid-term FU visit, with a mean FU of 4.4 years ± 0.8 (range, 2.0–6.3 years) after surgery. Demographic and clinical characteristics are summarized in Table 1. The mean age at surgery was 77.1 ± 9.1 years, and females represented the majority of the cohort (90.7%). FNF was the indication for surgery and all 54 patients were treated by HA. Osteoporosis was documented in the medical history of 10 patients (18.5%).

### 3.2. Surgical and Perioperative Characteristics

Most procedures were performed through a lateral approach (75.9%) under subarachnoid anaesthesia. Regarding preoperative characteristics, 26 patients (48.1%) were classified as ASA physical status II and 28 (51.9%) as ASA III (Table 2). The mean operative time was 96.7 ± 37.7 min (median, 92.5 min; IQR, 75.0–111.5 min; range, 35–265 min). Two prolonged procedures involved additional surgical interventions, including treatment of a concomitant humeral fracture during the same operative session (208 min) and osteosynthesis for an intraoperative femoral fracture (265 min). In a descriptive sensitivity analysis excluding these two cases, the mean operative time was 91.3 ± 25.5 min (median, 92.0 min; IQR, 74.5–106.3 min; range, 35–155 min; *n* = 52). One intraoperative femoral fracture (1.9%) was reported and was considered procedure-related but not implant-related. No intraoperative haemodynamic complications or clinically suspected cases of BCIS were reported. Intraoperatively, femoral bone stock was assessed as normal in 42 patients (77.8%) and porotic in 12 (22.2%). No cases were considered to have bone stock inadequate for implantation, and no bone grafting procedures were required (Table 2). The mean length of hospital stay was 11.4 ± 4.7 days (median, 9.5 days; IQR, 8–13). Perioperative blood transfusions were administered to 21 patients (38.9%). At discharge, 20 patients (37.0%) returned home, whereas 34 (63.0%) were transferred to intermediate care, inpatient rehabilitation, or residential care facilities (Table 2).

### 3.3. Implant Characteristics

All patients received the CL TRAUMA femoral stem. The medium stem size was the most frequently implanted (53.7%), followed by the small (38.9%) and large (7.4%) sizes (Table 2). All modular femoral heads featured a 12/14 taper and were selected according to patient anatomy and surgical indication. Cobalt–chromium–molybdenum (CoCrMo) was the most commonly used material (77.8%), followed by AISI 316L stainless steel (18.5%), and Biolox Delta ceramic (3.7%). All patients received a 28 mm femoral head. All patients received a Lock Self-Centering bipolar head, consisting of an AISI 316L outer shell and an ultra-high-molecular-weight polyethylene (UHMWPE) liner, with the outer diameter selected according to the native acetabular anatomy. The most frequently used bipolar head diameters ranged from 44 to 49 mm.

### 3.4. Clinical Assessment

Clinical outcomes at mid-term FU are summarized in Table 3.

The mean HHS was 80.6 ± 16.9, and 33 patients (61.1%; 95% CI: 47.8–73.0%) achieved the primary endpoint of satisfactory functional status (HHS ≥ 80). According to the HHS classification, 22 patients (40.7%) achieved excellent results, 11 (20.4%) good, 10 (18.5%) fair, 5 (9.3%) poor, and 6 (11.1%) were classified as failures.

At mid-term FU, gait assessment showed that 40 patients (74.1%) walked without a limp, while 10 (18.5%) had a slight limp, three (5.6%) a moderate limp, and one (1.9%) a severe limp. Twenty-four patients (44.4%) walked without assistive devices, and 47 (87.0%) reported a walking distance of at least two to three blocks (Table 3).

Mean patient satisfaction, assessed using the VAS, was 76.4 ± 29.3, with a median of 90 (IQR, 61.2–100). The mean ROM was 103.0 ± 14.4° for flexion, 35.0 ± 10.1° for abduction, and 23.7 ± 9.1° for external rotation. Most patients demonstrated extension <10° (86.8%), adduction ≤20° (83.3%), and internal rotation ≤20° (79.6%) at mid-term FU.

### 3.5. Radiographic Assessment

Preoperative radiographic findings are summarized in Table 4.

According to the Garden classification, 23 patients (42.6%) presented with Garden III fractures and 31 (57.4%) with Garden IV fractures. Most hips presented with neutral alignment (96.3%), while varus deformity was observed in 3.7% of cases. Osteophytes were present in 14.8% of cases, mainly involving the acetabulum (87.5%). No cystic lesions were detected. Sclerosis was observed in 83.3% of patients, most commonly involving the acetabulum (88.6%).

**Figure 3:**
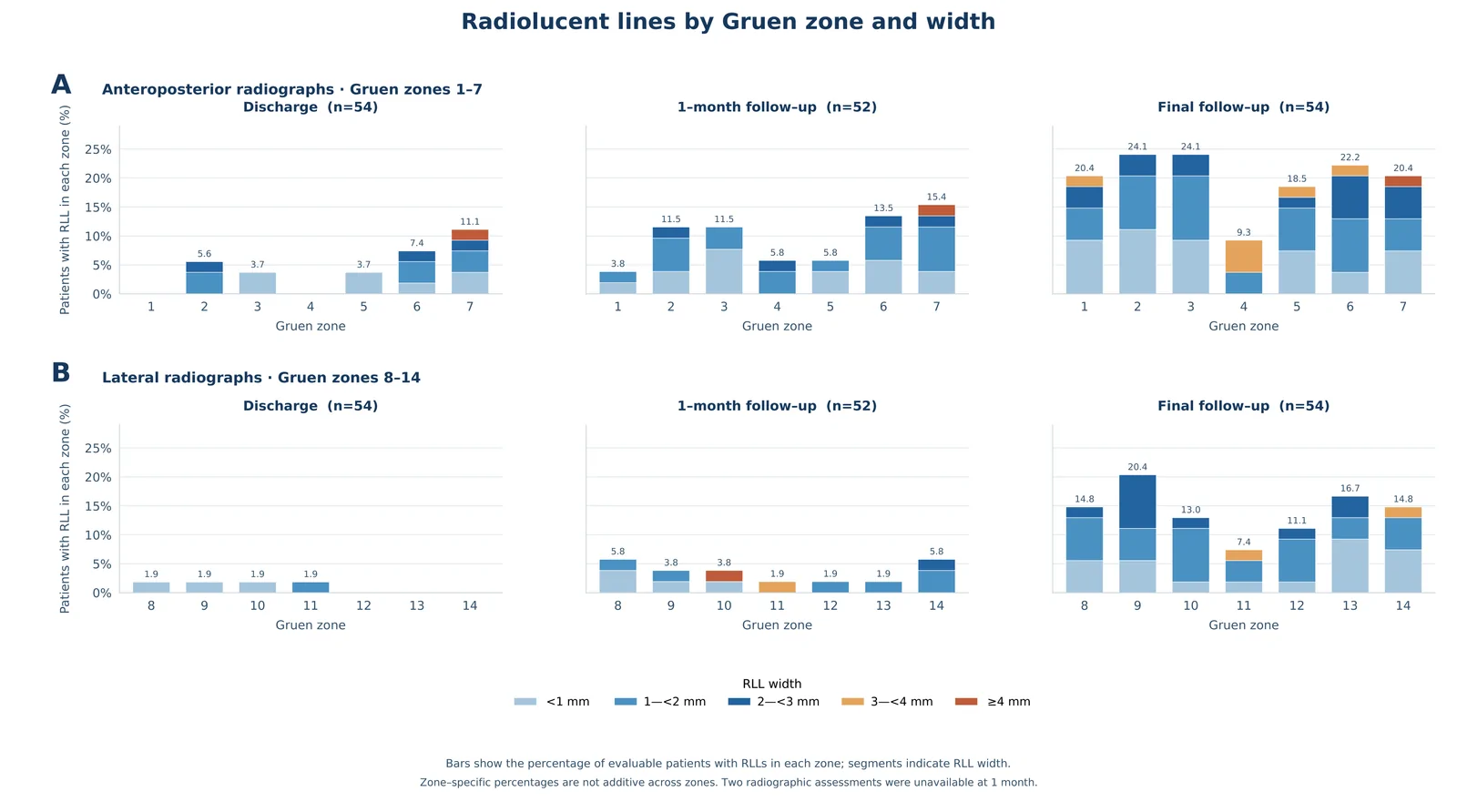
Distribution and thickness of radiolucent lines (RLLs) according to Gruen zones at different follow-up assessments. (**A**) Anteroposterior (AP) radiographs, Gruen zones 1–7; (**B**) lateral radiographs, Gruen zones 8–14. Stacked bars represent the percentage of patients with RLLs in each Gruen zone, stratified by thickness (<1 mm, ≥1 to <2 mm, ≥2 to <3 mm, ≥3 to <4 mm, and ≥4 mm). Radiographic assessments were available for 54 patients at discharge, 52 at the 1-month follow-up, and 54 at the final mid-term follow-up. Percentages were calculated using the number of patients with evaluable radiographs at each time point. As individual patients could exhibit RLLs in multiple Gruen zones, zone-specific percentages are not additive.

Postoperative radiographic evaluation demonstrated a progressive increase in the prevalence of RLLs, from 13.0% at discharge to 23.1% at the 1-month FU and 38.9% at mid-term FU. Accordingly, the proportion of patients without RLLs decreased from 87.0% to 61.1% during the same period. Most RLLs remained minor and stable throughout FU. The distribution and thickness of RLLs according to Gruen zones in AP and lateral projections at each radiographic time point are reported in Figure 3, Tables S1 and S2.

On AP radiographs, RLLs were most frequently observed in Gruen Zone 7 at discharge (11.1%) and at the 1-month FU (15.4%). At the mid-term FU, the highest frequencies were recorded in Zones 2 and 3 (24.1% each), followed by Zone 6 (22.2%) and Zones 1 and 7 (20.4% each). On LAT radiographs, RLLs were infrequent at discharge but became more frequently detectable at subsequent assessments. At the mid-term FU, they were most commonly observed in Zone 9 (20.4%), followed by Zone 13 (16.7%) and Zones 8 and 14 (14.8% each). Regarding RLL thickness, most radiolucencies measured less than 2 mm at the mid-term FU, accounting for 72.0% and 77.4% of affected Gruen-zone observations on AP and LAT radiographs, respectively. These findings indicate that most radiographic observations involved relatively thin RLLs, although wider radiolucencies were also documented in selected zones. Radiographs were unavailable for two patients at the 1-month FU, and the corresponding analyses were based on 52 evaluable patients.

All femoral stems showed neutral coronal alignment, with no varus or valgus malalignment observed.

Assessment of initial cement mantle quality on discharge radiographs showed Bar-rack grade A in 36 of 54 hips (66.7%) and grade B in 18 hips (33.3%). No grade C or D ce-ment mantles were identified on the initial postoperative radiographs. Longitudinal changes in the radiographic appearance of the cement–bone interface were evaluated separately through the analysis of RLLs according to the Gruen zones, as described above.

Regarding acetabular erosion, all 54 hips were classified as Baker grade 0 at dis-charge. At mid-term FU, 9 hips (16.7%) were classified as grade 0, 33 (61.1%) as grade 1, and 12 (22.2%) as grade 2. No grade 3 cases or protrusio acetabuli were identified.

**Figure 4:**
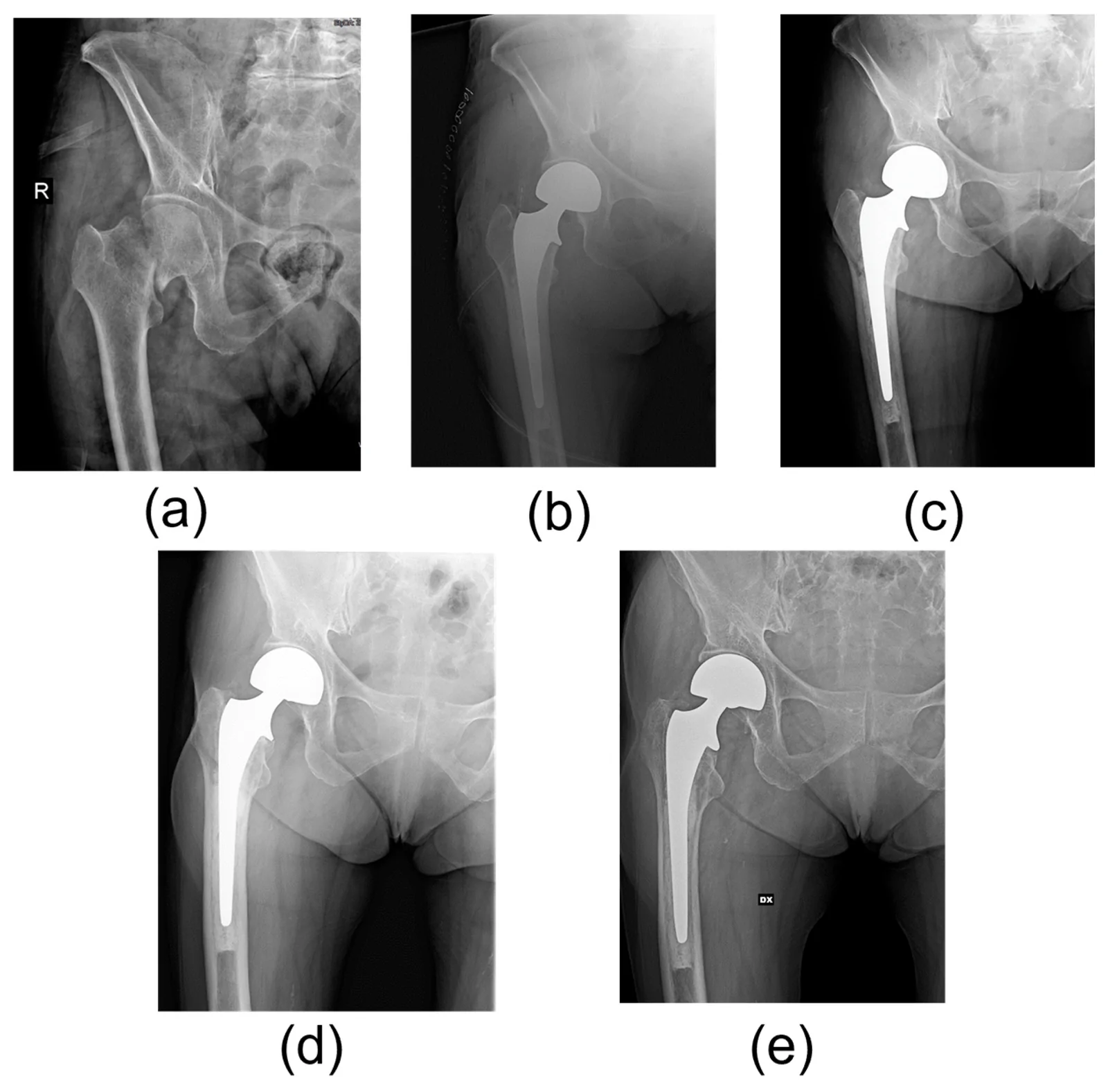
Anteroposterior radiographs of the right hip in an 85-year-old female patient treated with cemented hemiarthroplasty using the CL TRAUMA femoral stem for a displaced femoral neck fracture. (**a**) Preoperative radiograph; (**b**) Immediate postoperative radiograph; (**c**) 1-month follow-up; (**d**) 1-year follow-up; (**e**) Mid-term follow-up at 4 years, showing stable implant position and satisfactory radiographic appearance.

Distal stem migration/subsidence was detected in one patient (1.9%) showing a distal component migration of the femoral stem of 6 mm, not associated with the occurrence of major RLLs. Bone remodeling was observed in one patient (1.9%) at the 1-month follow-up and in three patients (5.6%) at mid-term FU. A representative case illustrating the radiographic evolution and implant stability at mid-term Follow-up is shown in Figure 4.

### 3.6. Safety Outcomes

A total of 11 SAEs were documented in 10 of the 54 enrolled patients (18.5%; 95% CI 10.2–31.0%) during the available observation period. None of these events were related to the investigational device, and none required device removal. Regarding their relationship to the study procedure, three SAEs (27.3%) were classified as related, two (18.2%) as possibly related, and six (54.5%) as unrelated.

Most SAEs were of moderate severity (8/11, 72.7%), while three (27.3%) were classified as severe. Seven events (63.6%) resulted in complete recovery, two (18.2%) in recovery with residual effects, one (9.1%) remained ongoing, and the outcome of one event (9.1%) was unknown. Regarding expectedness, five SAEs (45.5%) were classified as anticipated, none as unanticipated, and six (54.5%) as not applicable (Table 5).

Individual SAE descriptions, timing, seriousness criteria, severity, management, outcomes, expectedness, and causal relationships with the investigational device and study procedure are presented in Supplementary Table S3. These findings refer exclusively to the enrolled cohort, and the reported 18.5% should not be interpreted as the overall incidence of postoperative AEs in the original implanted population.

### 3.7. Exploratory Correlation Analyses

Spearman correlation analysis revealed a weak-to-moderate positive association between HHS and patient satisfaction (ρ = 0.38, 95% CI: 0.12–0.59; *p* = 0.005). In contrast, HHS showed weak negative correlations with age at surgery (ρ = −0.24, 95% CI: −0.48 to 0.02; *p* = 0.07) and BMI (ρ = −0.22, 95% CI: −0.47 to 0.07; *p* = 0.13), neither of which reached statistical significance.

## 4. Discussion

The present study evaluated the mid-term clinical, radiographic, and safety outcomes of the CL TRAUMA cemented femoral stem combined with a bipolar head in a cohort of patients undergoing HA for FNFs. While femoral component-related radiographic findings provide information on stem fixation and stability, clinical outcomes, patient satisfaction, range of motion (ROM), and overall safety reflect the performance of the hemiarthroplasty construct and cannot be attributed to the femoral stem alone. The main findings were satisfactory mid-term functional status, high patient satisfaction, limited radiographic changes, and the absence of device-related SAEs.

HA remains one of the most commonly adopted treatment options for displaced FNFs in elderly patients, particularly in frailer or lower-demand individuals, because it provides reliable restoration, while requiring a less demanding surgical procedure, shorter operative time, and lower costs than THA, thereby facilitating early mobilization [4,22]. When HA is indicated, many surgeons prefer bipolar constructs because they may reduce the risk of dislocation and potentially limit acetabular erosion compared with unipolar HA [23,24,25].

Current evidence supports the use of cemented femoral stem fixation in this setting, particularly in patients with compromised bone quality, owing to its superior mechanical stability and lower risk of periprosthetic fracture and revision surgery compared with uncemented stems [24,25,26,27,28]. Recent evidence further suggests that cemented fixation may be associated with a modest reduction in mortality without increasing perioperative risk [26]. Although the mechanisms underlying this association remain uncertain, improved primary implant stability and the resulting facilitation of early postoperative mobilization may contribute to reduced medical complications and improved survival following hip fracture surgery [26,29,30].

While concerns regarding BCIS remain, appropriate patient selection, multidisciplinary perioperative management, and adherence to modern cementing techniques have substantially reduced its clinical impact, supporting the continued use of cemented fixation in appropriately selected elderly patients [26]. Although the present study did not include a comparison group, the satisfactory clinical, radiographic, and safety outcomes are consistent with the available evidence supporting cemented fixation in elderly patients with displaced FNFs [26].

Large national arthroplasty registries provide additional context for these findings. Registry-based analyses consistently support the use of cemented femoral fixation in HA for displaced FNFs. In the American Joint Replacement Registry (AJRR), cementless fixation was associated with higher risks of all-cause revision and revision for periprosthetic fracture compared with cemented HA [31]. Similarly, analysis of 62,875 HAs from the Australian Orthopaedic Association National Joint Replacement Registry (AOANJRR) identified cemented fixation as protective against revision [32]. Data from the Swedish Arthroplasty Register further provide long-term benchmarks for revision after HA for FNF. In a recent analysis of 33,059 hemiarthroplasties, 10-year revision rates of 4.6% and 5.0% were reported for two established hemiarthroplasty head constructs [33]. More recent AJRR data reported an overall 5-year cumulative revision risk of 3.2% after arthroplasty for FNF [22]. These large-scale registry findings provide relevant context for the absence of femoral stem revisions observed among the 54 patients evaluated in the present study. However, direct comparison should be interpreted cautiously because registry analyses include substantially larger and less selected populations with systematic longitudinal FU, whereas the present findings are restricted to patients who survived, were contactable and attended the mid-term assessment.

At a mean FU of 4.4 ± 0.8 years, 61.1% of patients met the predefined HHS threshold (HHS ≥ 80), with a mean HHS of 80.6 ± 16.9. Functional outcomes following cemented HA have also been reported by Langslet et al. who observed a mean HHS of 76.3 at 5 years in a randomized trial involving elderly patients treated with a different cemented femoral stem design [34]. However, direct comparisons between these findings are limited by differences in study design, patient selection, and FU procedures. In particular, the present HHS estimate was obtained from a selected cohort representing only 11.4% of the original implanted population, comprising patients who survived and were able and willing to attend the mid-term FU assessment. This introduces potential survivor and selection bias, which may have led to an overestimation of functional outcomes relative to the overall implanted population.

Although 61.1% of patients achieved an HHS ≥ 80 at mid-term FU, functional outcomes were heterogeneous, with 18.5% classified as fair and 20.4% as poor or failure. Importantly, no formal performance success criterion was pre-specified; therefore, the proportion of patients achieving the primary endpoint should be interpreted descriptively rather than as evidence that a predefined device performance goal was met. These findings highlight the variability in functional recovery and warrant cautious interpretation, particularly considering the advanced age and comorbidity burden of the study population.

Patient satisfaction was also high (mean VAS score 76.4 ± 29.3), indicating that the clinical benefits achieved after surgery were obtained despite the advanced age and comorbidity burden of the study population. This observation is consistent with data from the Norwegian Hip Fracture Register, where patients undergoing bipolar HA reported greater postoperative satisfaction than those treated with internal fixation, supporting the ability of HA to achieve favorable patient-reported outcomes following displaced FNFs [35].

Radiographic evaluation revealed an increase in the prevalence of RLLs from 13.0% at discharge to 38.9% at mid-term FU. However, most RLLs were of limited thickness, with only two patients showing radiolucencies ≥ 3 mm. Initial cement mantle quality was classified as Barrack grade A or B in all patients, indicating the absence of major cementation defects on post-operative radiographs. A single case of femoral stem subsidence (6 mm) was documented, without associated major RLLs. Overall, these findings suggest that radiographic changes at the cement–bone interface occurred during FU, although their clinical significance remains uncertain. Moreover, the absence of intermediate radiographic assessments between 1 month and mid-term FU prevents a precise determination of the timing and progression of these changes. Importantly, no femoral stem revisions were documented among the 54 patients evaluated at mid-term FU [26].

Acetabular changes represent an additional relevant radiographic finding after bipolar HA. At mid-term FU, 61.1% of patients exhibited Baker grade 1 changes, characterized by narrowing of the acetabular joint space, while 22.2% showed grade 2 changes involving acetabular bone erosion. No grade 3 erosion or protrusio acetabuli was identified. In a study of 37 patients undergoing bipolar HA with a minimum follow-up of 10 years, Schiavi et al. reported Baker grade 0–1, and 2–3 changes in 64.9%, and 35.1% of patients, respectively [36]. These findings indicate that acetabular changes are relatively common in these cohorts. Importantly, acetabular changes reflect the articulation between the bipolar head and the native acetabulum and should be considered separately from femoral stem fixation. Their clinical significance remains to be established, particularly regarding their potential association with pain, functional outcomes, and patient satisfaction.

Regarding device-related safety, 11 serious AEs occurred in 10 patients (18.5%). None of these events was considered related to the CL TRAUMA femoral stem, and no adverse device effects were identified. However, three events were considered related and two possibly related to the surgical procedure. Therefore, the overall safety findings should be interpreted in the context of the entire hemiarthroplasty procedure and patient population rather than as a direct measure of the safety profile of the femoral stem alone. This incidence is consistent with published reports in elderly patients undergoing HA for FNFs, in whom postoperative complications are frequently attributable to advanced age, frailty, and multiple comorbidities rather than implant performance [26,37]. Previous studies evaluating cemented stems have primarily focused on major orthopedic complications, including dislocation, periprosthetic fracture, and revision surgery [1,26]. In the present cohort, no implant removals or revision procedures were documented during the available FU, and none of the recorded SAEs was considered related to the CL TRAUMA femoral stem.

These findings further support the importance of an integrated clinical and radiographic evaluation in orthopedic practice, particularly for the longitudinal assessment of implant-related findings and patient outcomes [38]. Previous studies on other hip arthroplasty systems have shown that implant FU provides clinically relevant information beyond conventional functional outcomes, particularly when radiographic findings and material-related safety aspects are considered [39,40]. Therefore, continued clinical and radiographic FU remains important for the assessment of both patient outcomes and implant-related findings over time.

The present finding is consistent with previous evidence suggesting that advanced age alone should not preclude satisfactory functional status following cemented hip arthroplasty [28]. Among the evaluated factors, a weak-to-moderate positive correlation between HHS and patient satisfaction suggests that patients reporting better hip-related function also tended to report greater satisfaction. However, given the limited sample size and exploratory nature of the analysis, this association should not be interpreted as evidence of a causal relationship.

The main strengths of this study include the use of standardized mid-term clinical, radiographic, and safety assessments at mid-term FU in a real-world post-market setting. In addition, the study reflects routine clinical practice in an elderly population consisting of patients undergoing HA for FNFs.

Nevertheless, some limitations should be acknowledged. The single-center design, retrospective identification of patients, relatively small sample size, and lack of a comparison group limit the strength of the conclusions. Furthermore, because all patients received a bipolar HA construct, clinical and patient-reported outcomes cannot be attributed specifically to the CL TRAUMA femoral stem, but reflect the combined effects of the implant construct, patient characteristics, surgical technique, perioperative management, and rehabilitation. Of the 474 patients who received the study stem, only 54 (11.4%) were included in the mid-term analysis because they were alive, contactable, willing, and physically able to attend the FU visit. Moreover, 236 of 474 patients (49.8%) had died before recruitment. This substantial attrition introduces important selection and survivor bias and limits the generalizability of the findings to the entire implanted population. Although no femoral stem revisions were documented among the 54 evaluated patients, this finding should not be interpreted as an estimate of implant survivorship because revision status was not systematically available for the 420 patients who were not enrolled. Therefore, implant survivorship and revision rates for the overall implanted population cannot be reliably estimated.

No formal performance success criterion was pre-specified for the primary endpoint; therefore, the proportion of patients achieving HHS ≥ 80 should be interpreted descriptively rather than as evidence that a predefined device performance goal was met. Moreover, HHS has not undergone comprehensive validation specifically in very elderly patients undergoing HA for FNFs. Nevertheless, it is a well-established and widely used clinical instrument for assessing hip-related pain and function following hip arthroplasty, including HA for FNFs. Osteoporosis was not systematically assessed by DXA in this cohort; therefore, the reported prevalence reflects previously documented diagnoses and may underestimate the true burden of osteoporosis in this elderly fracture population.

In addition, the functional assessment was performed at a variable mid-term FU without standardized serial functional measurements or information on pre-fracture functional status. Therefore, the study provides a cross-sectional assessment of mid-term function rather than the trajectory or magnitude of postoperative recovery. No intermediate radiographic assessments were available between the 1-month visit and the final mid-term FU. Consequently, the exact timing of the development or progression of RLLs, as well as the timing of the single case of distal stem migration, cannot be determined. Furthermore, as radiographs were obtained under routine clinical conditions, minor variations in magnification, patient positioning, limb rotation, and centring may have introduced variability in radiographic measurements. Safety analyses were restricted to documented SAEs among the 54 enrolled patients. Retrospective ascertainment of earlier events and the inclusion of only patients who survived and attended the mid-term assessment may have introduced under-ascertainment, recall bias, and survivor bias. Consequently, the observed SAE proportions cannot be extrapolated to the entire implanted population. Although dates of death were retrieved to assess overall patient survival, the causes of death were not systematically available, preventing an assessment of their potential relationship to the surgical procedure or implanted device. Furthermore, the advanced age and comorbidity burden of the study population (ASA physical status II-III), together with the high mortality observed in the original cohort, limited the availability of long-term clinical FU.

## 5. Conclusions

Among patients who survived and completed mid-term FU after HA with the CL TRAUMA cemented femoral stem and a bipolar head for FNFs, satisfactory functional status and patient satisfaction were observed, with no device-related SAEs or femoral stem revisions. These findings are descriptive and limited to the selected cohort evaluated and should not be interpreted as evidence of implant survivorship in the overall implanted population.

## Figures and Tables

**Table 1:** Demographic and clinical features of the overall cohort.

Parameter	Overall Cohort (*n* = 54)
Age at surgery, yrs	77.1 ± 9.1
Gender, *n* (%)	
Female Male	49 (90.7) 5 (9.3)
BMI *, kg/m^2^	24.2 ± 4.5
Physical activity *n* (%)	
Normal Sedentary	12 (22.2) 42 (77.8)
Affected side, *n* (%)	
Left Right	21 (38.9) 33 (61.1)
Previous treatment or surgery on the affected side, *n* (%)	
No Yes	52 (96.3) 2 (3.7)
Previous treatment or surgery on the contralateral side, *n* (%)	
No Yes	49 (90.7) 5 (9.3)
Recorded history of osteoporosis; *n* (%)	
No Yes	44 (81.5) 10 (18.5)

BMI: body mass index. Data are reported as mean ± standard deviation unless otherwise specified. * data were available for 48 patients.

**Table 2:** Perioperative and implant characteristics of the study cohort.

Parameter	Overall Cohort (*n* = 54)
**Preoperative characteristics**	
ASA physical status, *n* (%)	
II III	26 (48.1) 28 (51.9)
**Surgical characteristics**	
Surgical approach, *n* (%)	
Antero-lateral Lateral Postero-lateral	10 (18.5) 41 (75.9) 3 (5.6)
Anaesthesia, *n* (%)	
General Subarachnoid	1 (1.9) 53 (98.1)
Skin-to-skin operative time, min (*n* = 54)	
Mean ± SD; median (IQR); range	96.7 ± 37.7; 92.5 (75.0–111.5); 35–265
Sensitivity analysis, min (*n* = 52)	
Mean ± SD; median (IQR); range	91.3 ± 25.5; 92.0 (74.5–106.3); 35–155
Intraoperative complications, *n* (%)	
No Yes, not implant-related	53 (98.1) 1 (1.9)
Antibiotic prophylaxis, *n* (%)	
No Yes	0 (0) 54 (100)
Antithrombotic prophylaxis, *n* (%)	
No Yes	1 (1.9) 53 (98.1)
Intraoperatively assessed femoral bone stock quality, *n* (%)	
Normal Porotic	42 (77.8) 12 (22.2)
**Implant characteristics**	
Femoral stem size, *n* (%)	
Small Medium Large	21 (38.9) 29 (53.7) 4 (7.4)
Modular femoral head material, *n* (%)	
CoCrMo	42 (77.8)
AISI 316L	10 (18.5)
Biolox Delta	2 (3.7)
Femoral head diameter, *n* (%)	
28 mm	54 (100)
**Postoperative characteristics**	
Hospital length of stay (days)	
Mean ± SD; Median (IQR)	11.4 ± 4.7; 9.5 (8–13)
Perioperative blood transfusion, *n* (%)	
None 1 unit 2 unit	33 (61.1) 13 (24.1) 8 (14.8)
Discharge destination, *n* (%)	
Home Intermediate care facility Inpatient rehabilitation unit Residential care facility	20 (37.0) 24 (44.4) 9 (16.7) 1 (1.9)

ASA = American Society of Anesthesiologists; SD = standard deviation; IQR = interquartile range; CoCrMo = cobalt–chromium–molybdenum.

**Table 3:** Clinical outcomes at mid-term Follow-up visit.

Outcome	Overall Cohort (*n* = 54)
HHS	80.6 ± 16.9, 95% CI (76.1–85.1) Range (24–99)
Median, (IQR)	88, (73.5–93.8)
HHS rating, *n* (%)	
Excellent Good Fair Poor Failure	22 (40.7) 11 (20.4) 10 (18.5) 5 (9.3) 6 (11.1)
HHS ≥ 80, *n* (%; 95% CI)	33 (61.1; 47.8–73.0)
Gait assessment—Limp, *n* (%)	
No limp Slight limp Moderate limp Severe limp	40 (74.1) 10 (18.5) 3 (5.6) 1 (1.9)
Walking support, *n* (%)	
No support Cane for long walks Cane most of the time One crutch Two crutches or unable to walk Two canes	24 (44.4) 5 (9.3) 4 (7.4) 11 (20.4) 5 (9.3) 5 (9.3)
Walking distance, *n* (%)	
Unlimited	5 (9.3)
Six blocks	24 (44.4)
Two to three blocks	18 (33.3)
Indoors only	6 (11.1)
Bed or chair only	1 (1.9)
VAS satisfaction	76.4 ± 29.3
Median, (IQR)	90, (61.2–100)
Flexion (°)	103 ± 14.4
Extension *, *n* (%)	
<10° ≥10°	46 (86.8) 7 (13.2)
Abduction (°), mean ± SD	35 ± 10.1
Adduction, *n* (%)	
≤20° >20°	45 (83.3) 9 (16.7)
Internal Rotation, *n* (%)	
≤20° >20°	43 (79.6) 11 (20.4)
External Rotation (°)	23.7 ± 9.1

HHS = Harris Hip Score; VAS = Visual Analogue Scale. Data are reported as mean ± standard deviation unless otherwise specified. * data were available for 53 patients.

**Table 4:** Preoperative radiographic characteristics.

Preoperative Radiographic Characteristics	Overall Cohort (*n* = 54)
Femoral neck fracture classification (Garden), *n* (%)	
Garden III Garden IV	23 (42.6) 31 (57.4)
Deformity, *n* (%)	
Neutral Valgus Varus	52 (96.3) 0 (0) 2 (3.7)
Osteophytes, *n* (%)	
No Yes	46 (85.2) 8 (14.8)
Osteophyte’s location, *n* (%)	
Acetabulum Femoral neck	7 (87.5) 1 (12.5)
Cysts, *n* (%)	
No Yes	54 (100) 0 (0)
Sclerosis, *n* (%)	
No Slight Moderate Marked	9 (16.7) 30 (55.5) 13 (24.1) 2 (3.7)
Sclerosis’s location, *n* (%)	
Acetabulum Acetabulum and Femoral head Supra-acetabular	39 (88.6) 3 (6.8) 2 (4.6)

**Table 5:** Serious adverse events and their characteristics in the enrolled cohort.

Parameter	*n* (%)
Patients with ≥1 SAE (*n* = 54)	
No Yes	44 (81.5) 10 (18.5)
Severity of SAEs (*n* = 11)	
Moderate Severe	8 (72.7) 3 (27.3)
Treatment of SAEs (*n* = 11)	
None Non-pharmacological Pharmacological Combined Unknown	1 (9.1) 4 (36.4) 2 (18.2) 3 (27.3) 1 (9.1)
Outcome of SAEs (*n* = 11)	
Recovered Recovered with residual effects Ongoing Unknown	7 (63.6) 2 (18.2) 1 (9.1) 1 (9.1)
Relationship to investigational device (*n* = 11)	
Related Not related	0 (0.0) 11 (100.0)
Relationship to the study procedure (*n* = 11)	
Related Possible Not related	3 (27.3) 2 (18.2) 6 (54.5)
Expectedness (*n* = 11)	
Anticipated Unanticipated Not applicable	5 (45.5) 0 (0.0) 6 (54.5)
Device removal required (*n* = 11)	
Yes No	0 (0.0) 11 (100.0)

SAEs = Serious Adverse Events. Percentages for SAE characteristics were calculated using the total number of documented events (*n* = 11), whereas the proportion of patients experiencing at least one SAE was calculated using the enrolled cohort (*n* = 54). Eleven SAEs occurred in ten patients.

## Data Availability

The data that support the findings of this study are available from the corresponding author, [EB], upon reasonable request.

## Supplementary Materials

The following supporting information can be downloaded at: https://www.mdpi.com/article/10.3390/jcm15218640/s1, Supplementary Figure S1. Kaplan–Meier curve of patient survival in the overall implanted cohort (*n* = 474), Table S1: Radiolucent lines by Gruen zone on anteroposterior radiographs, Table S2: Radiolucent lines by Gruen zone on lateral radiographs, Table S3: Serious adverse events recorded during follow-up.

## Abbreviations

The following abbreviations are used in this manuscript:

FNFs	Femoral neck fractures
HA	Hemiarthroplasty
THA	Total hip arthroplasty
PMMA	Polymethylmethacrylate
FU	Follow-up
HHS	Harris Hip Score
VAS	Visual Analogue Scale
AP	Anteroposterior
LAT	Lateral
RLLs	Radiolucent lines
ROM	Range of Motion
AE	Adverse events
SAEs	Serious adverse events
ADEs	Adverse device effects
DDs	Device deficiencies
SADEs	Serious adverse device effects
USADEs	Unanticipated serious adverse device effects
UHMWPE	Ultra-high-molecular-weight polyethylene
CoCrMo	Cobalt–chromium–molybdenum
CI	Confidence Interval
BMI	Body Mass Index
BCIS	bone cement implantation syndrome
