# Supplementary material for: Mid-Term Clinical, Radiographic, and Safety Outcomes After Hemiarthroplasty with the CL TRAUMA Cemented Femoral Stem: A Post-Market Clinical Investigation
Source: J Clin Med. 2026 Nov 6;15(21):8640. doi: 10.3390/jcm15218640 (PMC13764356; doi:10.3390/jcm15218640)
Supplement: Supplementary file 1 [file jcm-15-08640-s001.zip › jcm-4576185-supplementary.pdf]

Figure S1. Kaplan–Meier curve of patient survival in the overall implanted cohort (n = 474). Estimated survival was 76.6% at 1 year (95% CI: 72.5–80.1%) and 53.2% at 5 years (95% CI: 48.4–57.8%).

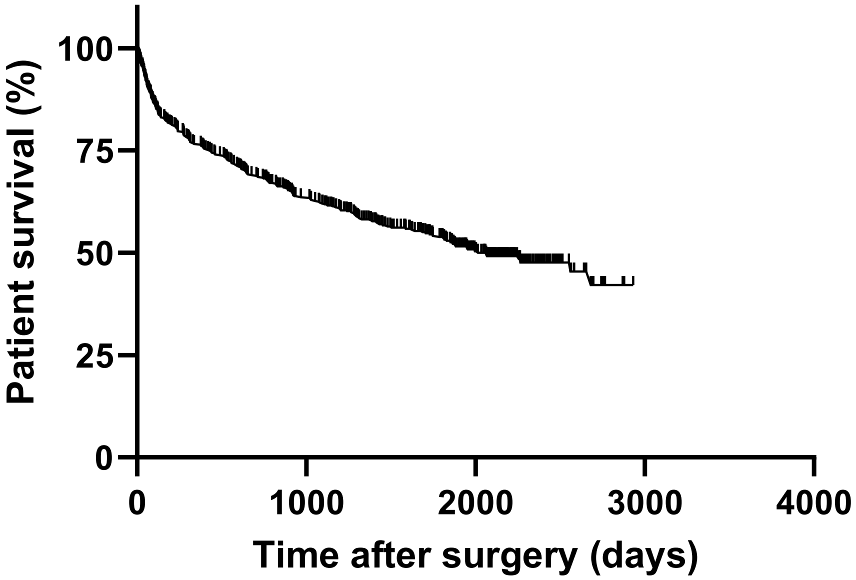

Table S1. Radiolucent lines by Gruen zone on anteroposterior radiographs

Time point / RLL thickness	Zone 1	Zone 2	Zone 3	Zone 4	Zone 5	Zone 6	Zone 7
**Discharge (N=54)**							
None	54 (100)	51 (94.4)	52 (96.3)	54 (100)	52 (96.3)	50 (92.6)	48 (88.9)
<1 mm	0 (0)	0 (0)	2 (3.7)	0 (0)	2 (3.7)	1 (1.9)	2 (3.7)
≥1 to <2 mm	0 (0)	2 (3.7)	0 (0)	0 (0)	0 (0)	2 (3.7)	2 (3.7)
≥2 to <3 mm	0 (0)	1 (1.9)	0 (0)	0 (0)	0 (0)	1 (1.9)	1 (1.9)
≥3 to <4 mm	0 (0)	0 (0)	0 (0)	0 (0)	0 (0)	0 (0)	0 (0)
≥4 mm	0 (0)	0 (0)	0 (0)	0 (0)	0 (0)	0 (0)	1 (1.9)
**1 month (N=52)**							
None	50 (96.2)	46 (88.5)	46 (88.5)	49 (94.2)	49 (94.2)	45 (86.5)	44 (84.6)
<1 mm	1 (1.9)	2 (3.8)	4 (7.7)	0 (0)	2 (3.8)	3 (5.8)	2 (3.8)
≥1 to <2 mm	1 (1.9)	3 (5.8)	2 (3.8)	2 (3.8)	1 (1.9)	3 (5.8)	4 (7.7)
≥2 to <3 mm	0 (0)	1 (1.9)	0 (0)	1 (1.9)	0 (0)	1 (1.9)	1 (1.9)
≥3 to <4 mm	0 (0)	0 (0)	0 (0)	0 (0)	0 (0)	0 (0)	0 (0)
≥4 mm	0 (0)	0 (0)	0 (0)	0 (0)	0 (0)	0 (0)	1 (1.9)
**Final follow-up (N=54)**							
None	43 (79.6)	41 (75.9)	41 (75.9)	49 (90.7)	44 (81.5)	42 (77.8)	43 (79.6)
<1 mm	5 (9.3)	6 (11.1)	5 (9.3)	0 (0)	4 (7.4)	2 (3.7)	4 (7.4)
≥1 to <2 mm	3 (5.6)	5 (9.3)	6 (11.1)	2 (3.7)	4 (7.4)	5 (9.3)	3 (5.6)
≥2 to <3 mm	2 (3.7)	2 (3.7)	2 (3.7)	0 (0)	1 (1.9)	4 (7.4)	3 (5.6)
≥3 to <4 mm	1 (1.9)	0 (0)	0 (0)	3 (5.6)	1 (1.9)	1 (1.9)	0 (0)
≥4 mm	0 (0)	0 (0)	0 (0)	0 (0)	0 (0)	0 (0)	1 (1.9)

Data are presented as n (%). RLL, radiolucent line. At the 1-month follow-up, radiographs were unavailable for two patients.

Table S2. Radiolucent lines by Gruen zone on lateral radiographs

Time point / RLL thickness	Zone 8	Zone 9	Zone 10	Zone 11	Zone 12	Zone 13	Zone 14
**Discharge (N=54)**							
None	53 (98.1)	53 (98.1)	53 (98.1)	53 (98.1)	54 (100)	54 (100)	54 (100)
<1 mm	1 (1.9)	1 (1.9)	1 (1.9)	0 (0)	0 (0)	0 (0)	0 (0)
≥1 to <2 mm	0 (0)	0 (0)	0 (0)	1 (1.9)	0 (0)	0 (0)	0 (0)
≥2 to <3 mm	0 (0)	0 (0)	0 (0)	0 (0)	0 (0)	0 (0)	0 (0)
≥3 to <4 mm	0 (0)	0 (0)	0 (0)	0 (0)	0 (0)	0 (0)	0 (0)
≥4 mm	0 (0)	0 (0)	0 (0)	0 (0)	0 (0)	0 (0)	0 (0)
**1 month (N=52)**							
None	49 (94.2)	50 (96.2)	50 (96.2)	51 (98.1)	51 (98.1)	51 (98.1)	49 (94.2)
<1 mm	2 (3.8)	1 (1.9)	1 (1.9)	0 (0)	0 (0)	0 (0)	0 (0)
≥1 to <2 mm	1 (1.9)	1 (1.9)	0 (0)	0 (0)	1 (1.9)	1 (1.9)	2 (3.8)
≥2 to <3 mm	0 (0)	0 (0)	0 (0)	0 (0)	0 (0)	0 (0)	1 (1.9)
≥3 to <4 mm	0 (0)	0 (0)	0 (0)	1 (1.9)	0 (0)	0 (0)	0 (0)
≥4 mm	0 (0)	0 (0)	1 (1.9)	0 (0)	0 (0)	0 (0)	0 (0)
**Final follow-up (N=54)**							
None	46 (85.2)	43 (79.6)	47 (87.0)	50 (92.6)	48 (88.9)	45 (83.3)	46 (85.2)
<1 mm	3 (5.6)	3 (5.6)	1 (1.9)	1 (1.9)	1 (1.9)	5 (9.3)	4 (7.4)
≥1 to <2 mm	4 (7.4)	3 (5.6)	5 (9.3)	2 (3.7)	4 (7.4)	2 (3.7)	3 (5.6)
≥2 to <3 mm	1 (1.9)	5 (9.3)	1 (1.9)	0 (0)	1 (1.9)	2 (3.7)	0 (0)
≥3 to <4 mm	0 (0)	0 (0)	0 (0)	1 (1.9)	0 (0)	0 (0)	1 (1.9)
≥4 mm	0 (0)	0 (0)	0 (0)	0 (0)	0 (0)	0 (0)	0 (0)

Data are presented as n (%). At the 1-month follow-up, radiographs were unavailable for two patients.

Table S3. Serious adverse events recorded during follow-up

Serious Adverse Event	Time to AE [months]	Seriousness criterion	Management	Outcome	Severity	Device causality	Procedure causality	Expectedness
Intraoperative femur fracture	0	Intervention required to prevent SAE	Osteosynthesis	Recovered	Moderate	Not Related	Related	Anticipated
Deep vein thrombosis	0.5	Intervention required to prevent SAE	Fogarty catheter procedure	Recovered	Severe	Not Related	Possible	Anticipated

Seroma at the wound site	1.2	Intervention required to prevent SAE	Surgical drainage	Recovered	Moderate	Not Related	Related	Anticipated
Renal Cancer	1.2	Life-threatening illness or Injury	Inoperable	Ongoing	Moderate	Not Related	Not Related	Not applicable
Pulmonary embolism	2.0	Hospitalization or prolongation of hospitalization	Caused by a deep vein thrombosis	Recovered with residual effect	Severe	Not Related	Possible	Anticipated
Periprosthetic calcifications	8.4	Intervention required to prevent SAE	Surgical excision of the calcifications	Recovered	Moderate	Not Related	Related	Anticipated
Minor stroke	12.0	Intervention required to prevent SAE		Unknown	Moderate	Not Related	Not Related	Not applicable
Primary hyperparathyroidism in plurinodular goiter	16.8	Intervention required to prevent SAE	Left parathyroidectomy	Recovered	Moderate	Not Related	Not Related	Not applicable
Vertebral fracture	44.4	Permanent impairment of body structure or function	Conservatively with a brace for about 60 days	Recovered	Severe	Not Related	Not Related	Not applicable
Atrial fibrillation	48.0	Life-threatening illness or Injury		Recovered with residual effect	Moderate	Not Related	Not Related	Not applicable
Bradycardia and Paroxysmal Atrial Fibrillation	52.8	Intervention required to prevent SAE	Dual-chamber pacemaker implantation followed by pharmacological treatment	Recovered	Moderate	Not Related	Not Related	Not applicable
